# Research on Enrichment of P_2_O_5_ from Low-Grade Carbonaceous Phosphate Ore via Organic Acid Solution

**DOI:** 10.1155/2019/9859580

**Published:** 2019-02-03

**Authors:** Fei Xie, Jie Zhang, Jiyan Chen, Jianrui Wang, Lin Wu

**Affiliations:** ^1^Mining College, Guizhou University, Guiyang 550025, China; ^2^Key Laboratory of Nonmetallic Mineral Resources Comprehensive Utilization, Guiyang 550025, China; ^3^Guizhou Engineering Lab of Mineral Resources, Guiyang 550025, China; ^4^National & Local Joint Laboratory of Engineering for Effective Utilization of Regional Mineral Resources from Karst Areas, Guiyang 550025, China; ^5^College of Resources and Environmental Engineering, Guizhou University, Guiyang 550025, China; ^6^Editorial Department of Journal, Guiyang University, Guiyang 550005, China; ^7^Bureau of Coal Geology of Guizhou Province, Guiyang 550008, China

## Abstract

The theory of using dilute organic acid solutions to leach the carbonaceous part from low-grade carbonaceous phosphate ore has been proposed by researchers as an effective approach to increase the proportion of P and to utilize the abundant low-grade resource. In this paper, a comprehensive experimental study was carried out to confirm the feasibility of organic acid leaching and investigate the optimized leaching conditions. Utilizing the low-grade carbonaceous phosphate ore produced in Zhijin, southwest of China, the effects of different types of acid, acid concentrations, reaction temperatures, reaction times, and liquid-solid ratios on leaching rate of P_2_O_5_ were evaluated using single-factor experiments and orthogonal experiments. The reaction mechanism, examined by SEM technique and the reaction thermodynamic analysis suggested that the leaching of P_2_O_5_ mainly resulted from the process of dissolution of dolomite (the main gangue mineral) in organic acid, consequently enriching the phosphate rock (the mineral of value). The effectiveness and impacts of different types of acid and reaction conditions were also studied. To conclude, this study first confirmed the viability of enriching P_2_O_5_ from low-grade ores through organic acid leaching the carbonaceous part by experimental data, and the experimental results will provide an essential scientific support for further upgrade of the technology to commercial scale utilization.

## 1. Introduction

Although the phosphate resources in China are abundant, they mainly consist of low-grade phosphate rock. The beneficiation of low-grade phosphate ores is also a worldwide problem, and effective technologies to process such ores are yet to be developed. As a result, most low-grade phosphate rocks are currently unexploited. Low-grade phosphate rock exploitation has become one of the main topics in mineral research, with emphasis on developing a cheap and environmentally friendly method to take full advantage of such phosphate resources [1, 2]. In China, most of the low-grade phosphate mines obtain phosphorus concentrate through the beneficiation process to proceed follow-up production, and a few of them are used to produce phosphate rock powder as fertilizer after activation (Li et al., 2015) [3–5]. Depending on different factors including ore types, gangue mineral characteristics, as well as the dissemination relationship [8], flotation, dense medium beneficiation, roasting, calcination, washing, etc., are usually employed to beneficiate and process sedimentary phosphorous deposits. In the process of phosphorus enrichment, the low-grade phosphate ore is often calcined by a type of thermochemical beneficiation process, or leached by a strong acid such as sulfuric acid, hydrochloric acid, or phosphoric acid [9]. However, the current methods are not very economically efficient and environmentally friendly.

Acid leaching of phosphate rock was first proposed in 1970s [10, 11], in the hope of avoiding beneficiation losses. In 1990s, some researchers begun to investigate the technique of leaching phosphate rock via organic acids solution [12–14], and since then some researchers have further studied approaches to utilize the low-grade ores [15–17]. Organic acids show an appreciable degree of selective leaching of calcareous material in low-grade phosphate ores and are proved to be effective and economical for the beneficiation of low-grade calcareous phosphate ores. Organic acid leaching is supposed to be very selective, environmentally friendly, and is able to generate products of high purity. However, the performance of this approach has not been comprehensively assessed by experimental studies, and hence its economic feasibility is not well understood. Gharabaghi et al. [17] also suggested that research studies about the leaching characteristics of specific phosphate ores and optimization of organic acid leaching techniques are needed.

In this study, organic acid was used as a leaching agent for dolomite gangue minerals to enrich low-grade phosphorite. Single-factor experiments and orthogonal experiments were carried out to investigate main influence factors on enrichment of low-grade phosphate rock. Then, the mechanism of organic acid leaching and reaction thermodynamic was discussed to explain organic acid selectivity when leaching phosphate. The aim of this study is to confirm the viability of application of organic acid leaching in processing low-grade phosphorite and provide scientific support to develop a new effective method to utilize low-grade phosphate resources.

## 2. Materials and Methods

### 2.1. Materials

The sample was obtained from Zhijin Xinhua Phosphate Rock in Guizhou Province, China. The sample is sedimentary rock, and the sample number is GL2-3#. The results of the chemical analysis of the sample are listed in Table 1. The chemical composition of the sample is 17.45% P_2_O_5_, 10.55% MgO, 889.76 × 10^−4^% ΣREE (total content of rare earth elements), and not particularly high levels of SiO_2_ and Al_2_O_3_.

There are three main types of phosphate rock in China, namely, magmatic phosphate rock, sedimentary phosphate rock, and sedimentary metamorphic phosphate rock, while 70% to 80% of which is sedimentary phosphate rock deposits. The composition of GL2-3# is typical of low-grade sedimentary dolomitic sedimentary phosphate rock containing rare earth. Due to the low phosphorus content in the rock, it is usually necessary to increase the content of P_2_O_5_ above to 25% by calcination or strong acid leaching before it can be utilized.

The citric acid, lactic acid, acetic acid, and other reagents used in this experiment were all analytical reagents.

### 2.2. Analysis

The chemical composition of the sample was analyzed using X-ray fluorescence spectroscopy (XRF) (Axios mAx4KW, Panalytical, Netherlands) through melt samples. The microstructure of the phosphate rock's surface was observed using scanning electron microscopy with an energy spectrometer (SEM-EDAX) (S-3400N, Hitachi, Japan).

### 2.3. Experiment Methods

Firstly, the sample was ground to 0.075 mm after drying at 105°C. Secondly, the water bath pot was heated to a preset temperature; about 10 g of the sample was poured slowly into a 500 cm^3^ beaker containing a certain concentration of dilute organic acid. Thirdly, the magnetic stirrer was started. After the sample reacted completely, the beaker was taken out, cooled, and filtered; the residue was washed, dried, and weighed.

The content of P_2_O_5_ in phosphate rock was determined by the ammonium phosphomolybdate volumetric method. This method was specified by the national standard of China (GB 223.61-88 Steel and alloy chemical analysis methods), which is suitable for determining the content of P_2_O_5_ of phosphate rock when it is above 0.5%.

The main operating parameters of dilute organic acid leaching are organic acid concentration, reaction temperature, and reaction time. The effect of stirring intensity on leaching was not so significant that it was not investigated in this study [21]. The stirring intensity was fixed at 300 rpm to ensure no sediment at the bottom of beaker. The parameter of liquid-solid ratio is tested by a single-factor experiment.

## 3. Results and Discussion

### 3.1. Results

The organic acids, as reported, are fully miscible with water and can be easily separated from the beneficiated solid phosphate product by filtration. It is reported that by dilution of the acid with water, the contact surface area between the dolomite and the acid increases [22]. Highly concentrated organic acid solution does not react with calcium and magnesium carbonate because of the high polarity of the O-H bond of the acid molecules. Also, it is necessary to use dilute solution for an effective reaction. In dilute solutions, water molecules tend to decrease the effect of polarity of the organic acids O-H bond [23]. Therefore, the research object of this paper was focused on dilute organic acid.

Many types of organic acids had been reported to be used for the leaching of phosphate rock [24]. Among them, acetic acid had been proved to enhance dissolution of calcium carbonate [25], while other types of organic acid had also been proved to have the ability to dissolve the carbonate materials in low-grade phosphate rock, such as oxalic, citric, lactic, malic, succinic, and formic acids [15, 26–28]. The research studies above indicate that the effect of the enrichment rate of P_2_O_5_ is closely related to the type of organic acid. In this article, three types of acid had been chosen, listed in Table 2, to investigate the impact factors of acid type and other leaching conditions.

#### 3.1.1. Single-Factor Experiments

In dilute organic acids, acid concentration is one of the key factors in achieving good results in the selective removal of dolomite mineral in low-grade phosphate rocks.  Table 3 presents the results obtained from leaching tests under different concentrations of the three organic acids at room temperature. The results demonstrate that the three types of organic acids tested showed good selectivity for the leaching of dolomite. All three organic acids can improve the grade of P_2_O_5_, from the low level, which is 12% to 25%, to the higher level, which is above 30%. It is proved that the dilute organic acid is a promising leaching agent for the enrichment of P_2_O_5_ in low-grade phosphate ore. And it seems that the weaker the acidity, the higher the ability to improve the grade.

**Figure 1:**
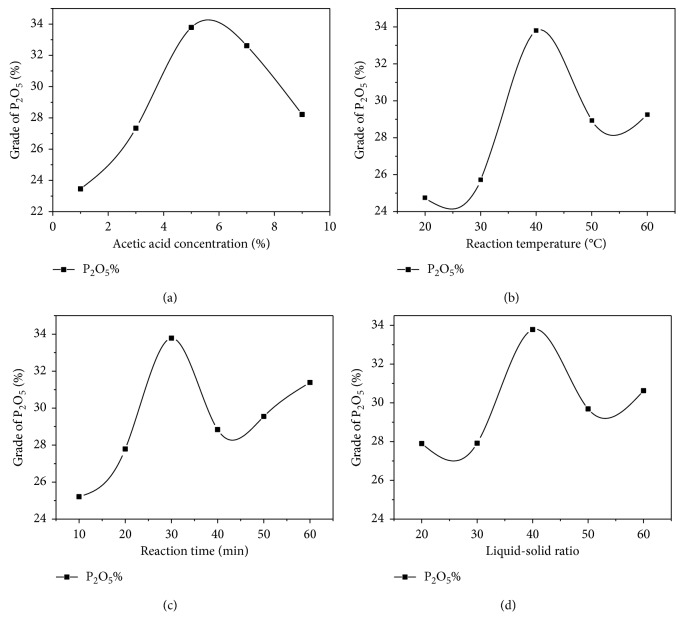
Results of single-factor experiments (a) L/S = 40 : 1(vol./wt.), *D* = 0.075 mm, at 40°C; (b) L/S = 40 : 1(vol./wt.), 5% acetic acid; (c) 5% acetic acid, at 40°C; (d) 5% acetic acid, at 40°C, *D* = 0.075 mm.

Furthermore, the effects of acid concentration, reaction temperature, reaction time, and liquid-solid ratio on the leaching rate of P_2_O_5_ were investigated using acetic acid as leaching reagent. Results are shown in Figure 1.

As seen in Figure 1(a), at the beginning, the grade of P_2_O_5_ increases along with the intensity of acid concentration. When the concentration of acetic acid is 5%, the grade of P_2_O_5_ reaches to the maximum value.

As shown in Figure 1(b), raising reaction temperature can increase the grade of P_2_O_5_. When the temperature is higher than 40°C, the dissolution of dolomite does not increase. This indicates that a higher temperature can increase the evaporation of water and acetic acid.

The P_2_O_5_ grade varies with the reaction time as shown in Figure 1(c). Firstly, as the leaching time lengthened, the P_2_O_5_ grade increases rapidly. At 30 minutes, the P_2_O_5_ rises up to 33.78%. After 30 minutes, the grade of P_2_O_5_ tends to fall and then rise but is lower than the value at 30 minutes. Within 60 minutes of the reaction, the grade of P_2_O_5_ experienced two increases; this phenomenon may indicate the following reaction. During the first 30 minutes, acetic acid selectively reacts with the exposed carbonate minerals in the sample particles. At the 30th minute, the first exposed carbonate minerals completely reacted with the acid. During the 30–40 minutes, the sample particles continued to dissolve in the acid solution, and some of the phosphorus-containing minerals were corroded by the solution, resulting in a decrease in the P_2_O_5_ grade. However, as time progressed, more carbonate mineral surfaces were exposed, and the acid in the solution reacted with it, resulting in an increase in P_2_O_5_ grade in 40–60 minutes.

As seen in Figure 1(d), it is found that the grade of P_2_O_5_ reaches the maximum value when liquid-solid ratio is 40 : 1(vol./wt.). When the liquid-solid ratio is low, the concentration of insoluble substance in the reaction becomes larger, and the insoluble substance is easily attached to the surface of the mineral to prevent hydrogen ions from further participating in the reaction. If the pulp is viscous, minerals do not fully react with acetic acid. Thus, the optimum liquid-solid ratio is 40 : 1(vol./wt).

#### 3.1.2. Orthogonal Experiments

Orthogonal experiments were designed to explore for effectiveness of different operating factors. The factors acid type, reaction temperature, acid concentration, and reaction time are listed in Table 4, while the L/S is 40 : 1(vol./wt). So, the L_9_ (3^4^) orthogonal experiments [29] were selected, and the results are shown in Tables 5 and 6.

Table 6 shows that the *R* value of factor A (acid type) is the biggest, and the acid type has the most significant effect on the leaching. The *R* values of the other factors are A > D > C > B, which determine the effects of different operating factors on the P_2_O_5_ grade as follows:

(1) $$\begin{array}{c} \text{acid type}>\text{reaction time}>\text{acid concentration}>\text{reaction temperature}. \end{array}$$

The range analysis shows that the optimum condition is A_3_B_3_C_3_D_2_, which is 11% lactic acid concentration, 55°C temperature, and 120 min reaction time. From Table 6, it seems that the stronger acid has the higher grade of P_2_O_5_. Therefore, this result together with the result of the single-factor experiments indicates that more studies should be conducted to reveal the influence on the type of organic acid of the P_2_O_5_ enrichment effect.

### 3.2. Discussion

#### 3.2.1. Mechanism of Organic Acid Leaching

Take citric acid as an example to illustrate the reaction process. Under conditions of weak acidity, because the hydrophilicity of dolomite is higher than apatite, dolomite is decomposed by citric acid first. The main chemical reaction occurring in the leaching process between dolomite (CaMg(CO_3_)_2_) and citric acid is [30–32]

(2) $$\begin{array}{c} {4\text{C}}_{6}{\text{H}}_{8}{\text{O}}_{7}\left(\text{aq}\right)+3\text{CaMg}{\left({\text{CO}}_{3}\right)}_{2}\left(\text{s}\right)={\text{Mg}}_{3}{\left({\text{C}}_{6}{\text{H}}_{5}{\text{O}}_{7}\right)}_{2}\left(\text{aq}\right)+{\text{Ca}}_{3}{\left({\text{C}}_{6}{\text{H}}_{5}{\text{O}}_{7}\right)}_{2}\left(\text{aq}\right)+{6\text{CO}}_{2}\left(\text{g}\right)+{6\text{H}}_{2}\text{O}\left(\text{l}\right) \end{array}$$

**Figure 2:**
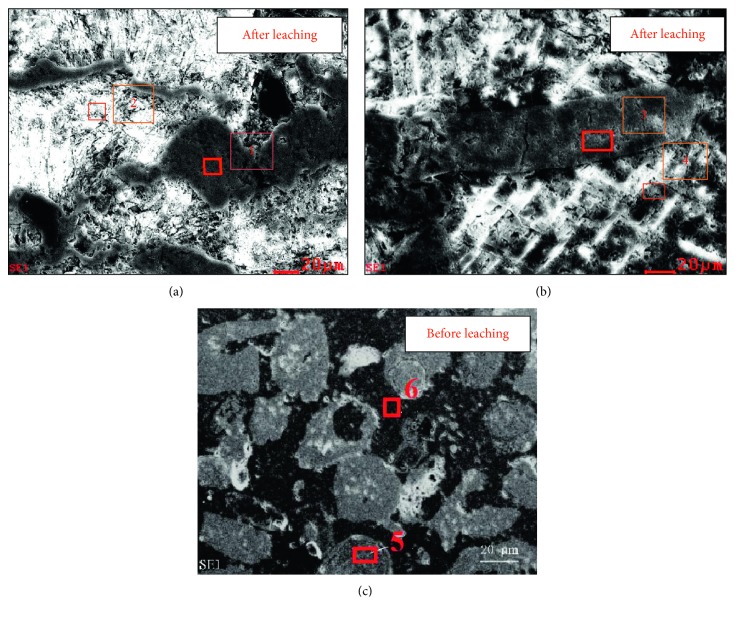
SEM-EDAX diagrams of phosphate rock after (a, b) and before (c) citric acid leaching. (Point 1, Point 3, and Point 6 are colloidal phosphate; Point 2, Point 4, and Point 5 are dolomite).

In order to characterize the reaction between dolomite and citric acid, SEM-EDAX was used to analyze the thin sections of phosphate rock. The results are shown in Figure 2 and Table 7.

It can be seen from Figure 2, before acid leaching, that the dolomite with the phosphate rock had a relatively smooth interface, and the colloidal phosphate was embedded between the dolomites' gaps. The interface between them was not clear. The microscopic appearance of the dolomite after leaching had undergone a great change, and the original smooth surface was dissolved by acid, revealing a clear texture. At the same time, the phosphate rock maintained its original smooth surface, presenting a clear interface with the dolomite. It shows that citric acid can dissolve dolomite and dissociate dolomite from colloidal phosphate. The particle volumes of colloidal phosphate had got a little change during the leaching process, indicating that the citric acid showed good selectivity with dolomite and colloidal phosphate.

#### 3.2.2. Dissolution Thermodynamic Analysis

In order to judge the thermodynamic process direction of the interaction between organic acid (e.g., acetic acid) and phosphate rock, the main method used at present is the minimum free energy method to study the important thermodynamic parameter of Gibbs free energy change (ΔG). If Gibbs free energy of the reaction system is decreased, namely, negative Gibbs free energy change, the reaction would occur automatically. The larger the negative value, the easier the reaction is to proceed completely.

The Gibbs free energy of the reaction in the standard state is

(3) $$\begin{array}{c} \Delta G^{\theta }=\Delta H^{\theta }-T\Delta S^{\theta }, \end{array}$$

where Δ*G* ^*θ*^ is the Gibbs free energy change in the standard state; Δ*H* ^*θ*^ is the enthalpy change in the standard state; Δ*S* ^*θ*^ is the entropy change in the standard state; and *T* is the thermodynamic temperature.

In the experiment of leaching phosphate rock by acetic acid, the following formula is recommended for the calculation of reaction kinetics as an example for the discussion of the thermodynamic process direction of the interaction:

(4) $$\begin{array}{c} 4{\text{CH}}_{3}\text{COOH}\left(\text{aq}\right)+\text{CaMg}\left({\text{CO}}_{3}\right)2\left(\text{s}\right)=2{\text{H}}_{2}\text{O}\left(\text{l}\right)+2{\text{CO}}_{2}\left(\text{g}\right)+\text{Ca}{\left({\text{C}}_{2}{\text{H}}_{3}{\text{O}}_{2}\right)}_{2}\left(\text{aq}\right)+\text{Mg}{\left({\text{CH}}_{3}\text{COO}\right)}_{2}\left(\text{aq}\right)\\ 10{\text{CH}}_{3}\text{COOH}\left(\text{aq}\right)+{\text{Ca}}_{5}{\left({\text{PO}}_{4}\right)}_{3}\text{F}\left(\text{s}\right)=5\text{Ca}{\left({\text{C}}_{2}{\text{H}}_{3}{\text{O}}_{2}\right)}_{2}\left(\text{aq}\right)+3{\text{H}}_{3}{\text{PO}}_{4}\left(\text{l}\right)+\text{HF}\left(\text{aq}\right) \end{array}$$

The results listed in Table 8 were carried out by the software HSC Chemistry v6.0. In the temperature range of 30°C to 70°C, Δ*G* ^*θ*^ of the reaction of acetic acid and dolomite is negative (Table 8). Thus, the reaction of acetic acid and dolomite can spontaneously proceed to the positive reaction direction. But, under the same circumstance, Δ*G* ^*θ*^ of the reaction of acetic acid and fluorapatite is positive (Table 9). This explains why the reaction of acetic acid and fluorapatite cannot spontaneously proceed to the positive reaction direction. Once more, this indicates that dolomite reacts with acetic acid prior to apatite.

## 4. Conclusions

It has been proposed by previous researchers that the grade of P_2_O_5_ of low-grade phosphate ore increases by leaching with organic acid. In this study, three organic acids have been studied through single-factor experiments and orthogonal experiments. The following conclusions can be drawn:

1. Lactic, citric, and acetic acids all have the ability to improve the grade of P_2_O_5_ from a low level to a high level, which is above 30%.
2. Acetic acid is more suitable for low temperature and fast, low concentration leaching, and the optimum parameters are 5% acid concentration, 40°C reaction temperature, and 30 min reaction time.
3. Orthogonal experiments show that the impact order of various factors on enrichment of low-grade phosphate rock is organic acid type > reaction time > acid concentration > reaction temperature. And the best conditions are lactic acid concentration at 11%, reaction temperatures at 55°C, and reaction time at 120 min.
4. SEM observations indicate that dolomite reacts with organic acid prior to apatite; the analysis results using thermodynamic theory also prove this.
5. The unconformity with the results of single-factor experiments and orthogonal tests indicates that more studies should be conducted to reveal the influence on the type of organic acid of the P_2_O_5_ enrichment effect.

## Figures and Tables

**Table 1:** Chemical analysis of phosphate rock (GL2-3#).

Chemical composition	Al_2_O_3_	CaO	Fe_2_O_3_	K_2_O	MgO	P_2_O_5_	SiO_2_	LOI	Others	REE
Content (%)	0.20	39.60	0.37	0.08	10.55	17.45	5.97	24.27	1.51	889.76 × 10^−4^

**Table 2:** The types of organic acid studied in the article.

Type of organic acid	pKa
Citric acid	3.13
Lactic acid	3.86
Acetic acid	4.76

**Table 3:** The leaching results of three kinds of organic acids at different acid concentrations.

Acid concentration (%)	Acid type		
	Grade of P_2_O_5_ (%)		
	Citric acid	Lactic acid	Acetic acid
1	17.72	18.94	23.46
3	20.66	22.87	27.34
5	23.58	24.14	33.78
7	25.46	28.42	32.62
9	27.36	28.88	28.22

**Table 4:** The operating factors of orthogonal experiments.

Code	Factors' name	Level 1	Level 2	Level 3
A	Acid type	Acetic acid	Citric acid	Lactic acid
B	Temperature (°C)	35	45	55
C	Concentration (%)	7	9	11
D	Reaction time (min)	60	120	180

**Table 5:** The results of orthogonal experiments.

Level	Factor				
	A	B	C	D	Index
	Acid type	Temperature (°C)	Concentration (%)	Reaction time (min)	P_2_O_5_ (%)
1	Acetic	35	7	60	28.43
2	Acetic	45	9	120	31.05
3	Acetic	55	11	180	32.43
4	Citric	35	9	180	32.75
5	Citric	45	11	60	31.74
6	Citric	55	7	120	32.83
7	Lactic	35	11	120	34.19
8	Lactic	45	7	180	32.54
9	Lactic	55	9	60	31.79

**Table 6:** The range analysis of P_2_O_5_ grade.

Factor	Level			
	K_1_	K_2_	K_3_	Range analysis
Acid type	91.91	97.32	98.52	6.61
Temperature	95.37	95.33	97.05	1.72
Concentration	93.80	96.04	96.71	2.91
Reaction time	91.96	98.07	97.72	6.11

**Table 7:** Spectral analysis of phosphate rock after citric acid leaching.

Content (%)	Element				
	C	O	Mg	P	Ca
1 wt.	8.40	20.60	0.37	18.45	52.17
2 wt.	18.41	33.82	10.26	—	37.51
3 wt.	—	23.45	—	20.74	55.80
4 wt.	9.64	38.62	12.02	—	39.71
5 wt.	19.97	44.53	15.77	—	19.72
6 wt.	27.50	34.10	—	24.24	14.15

**Table 8:** Δ*H* ^*θ*^, Δ*S* ^*θ*^, and Δ*G* ^*θ*^ of the reaction of acetic acid and dolomite.

Temperature (^o^C)	Δ*H* ^θ^ (kJ/mol)	Δ*S* ^*θ*^ (J/mol·K)	Δ*G* ^*θ*^ (kJ/mol)
30	−60.598	−144.752	−16.716
40	−45.053	−94.292	−15.525
50	−30.436	−48.339	−14.815
60	−16.582	−6.112	−14.546
70	−3.302	33.166	−14.683

**Table 9:** Δ*H* ^*θ*^, Δ*S* ^*θ*^, and Δ*G* ^*θ*^ of the reaction of acetic acid and fluorapatite.

Temperature (^o^C)	Δ*H* ^*θ*^ (kJ/mol)	Δ*S* ^*θ*^ (J/mol·K)	Δ*G* ^*θ*^ (kJ/mol)
30	52.182	−806.722	296.739
40	89.352	−686.064	304.193
50	124.269	−576.288	310.496
60	157.334	−475.505	315.748
70	189.003	−381.836	320.030

## Data Availability

All data included in this study are available upon request by contacting the corresponding author.

## Supplementary Materials

Supplementary 1 This file is the result file for thermodynamic calculation with HSC Chemistry v6.0. Readers can use the software and the chemical equation provided in this document to calculate the results.

Supplementary 2 This file is the sample chemical element analysis file provided by ALS Chemex company. This file provides the content information of most trace elements and most constant elements. In this paper, only part of the data information of “gl2-3 (raw ore)” sample in this file is used.
