# Supplementary material for: Research on Enrichment of P2O5 from Low-Grade Carbonaceous Phosphate Ore via Organic Acid Solution
Source: J Anal Methods Chem. 2019 Feb 3;2019:9859580. doi: 10.1155/2019/9859580 (PMC6377978; doi:10.1155/2019/9859580)
Supplement: Supplementary 1 — This file is the result file for thermodynamic calculation with HSC Chemistry v6.0. Readers can use the software and the chemical equation provided in this document to calculate the results. [file 9859580.f1.docx]

4CH3COOH(a) + CaMg(CO3)2(O) = 2H2O + 2CO2(g) + Ca(C2H3O2)2(a) + Mg(CH3COO)2(a)

T deltaH deltaS deltaG K Log(K)

C kJ J/K kJ

30.000 -60.598 -144.752 -16.716 7.595E+002 2.881

40.000 -45.053 -94.292 -15.525 3.889E+002 2.590

50.000 -30.436 -48.339 -14.815 2.483E+002 2.395

60.000 -16.582 -6.112 -14.546 1.909E+002 2.281

70.000 -3.302 33.166 -14.683 1.719E+002 2.235

Formula FM Conc. Amount Amount Volume

g/mol wt-% mol g l or ml

CH3COOH(a) 60.052 56.571 4.000 240.210 0.000 ml

CaMg(CO3)2(O) 184.403 43.429 1.000 184.403 0.000 ml

g/mol wt-% mol g l or ml

H2O 18.015 8.485 2.000 36.030 39.292 ml

CO2(g) 44.010 20.729 2.000 88.020 44.827 l

Ca(C2H3O2)2(a) 158.169 37.250 1.000 158.169 0.000 ml

Mg(CH3COO)2(a) 142.394 33.535 1.000 142.394 0.000 ml

10CH3COOH(a) + Ca5(PO4)3F = 5Ca(C2H3O2)2(a) +3H3PO4(l) + HF(g)

T deltaH deltaS deltaG K Log(K)

C kJ J/K kJ

30.000 52.182 -806.722 296.739 7.340E-052 -51.134

40.000 89.352 -686.064 304.193 1.800E-051 -50.745

50.000 124.269 -576.288 310.496 6.405E-051 -50.193

60.000 157.334 -475.505 315.748 3.088E-050 -49.510

70.000 189.003 -381.836 320.030 1.908E-049 -48.719

Formula FM Conc. Amount Amount Volume

g/mol wt-% mol g l or ml

CH3COOH(a) 60.052 54.354 10.000 600.524 0.000 ml

Ca5(PO4)3F 504.312 45.646 1.000 504.312 0.000 ml

g/mol wt-% mol g l or ml

Ca(C2H3O2)2(a) 158.169 71.580 5.000 790.845 0.000 ml

H3PO4(l) 97.995 26.609 3.000 293.985 0.000 ml

HF(g) 20.006 1.811 1.000 20.006 22.414 l
